# SOBA UK response to “emergencies in obese patients: a narrative review”

**DOI:** 10.1186/s44158-021-00027-2

**Published:** 2021-12-14

**Authors:** Andrew McKechnie, Laura Pengelly, John Cousins, on behalf of SOBA UK

**Affiliations:** 1 Lewisham and Greenwich NHS Trust, London, UK; 2 Northern General Hospital, Sheffield, UK; 3 St Marys, Imperial NHS Trust, London, UK

**Keywords:** Anaesthesia, Obesity, Emergency management

The letter supports the authors of the review and agrees with the need for improved guidance and organisational preparedness in this field

Dear Editor,

SOBA UK read this article with interest and welcome the review of managing patients with obesity in the emergency setting [1].

Many of the practical issues highlighted are common to non-emergent practice, but have the additional time pressure found in the emergency scenario. We would suggest that preparation and good situational awareness is the key to success in this patient group. Discussing the ‘Plan A’ and when to move to ‘Plan B’ for procedural skills can help teams to have the correct equipment available, and may help to avoid task fixation which can be an issue when standard techniques are failing in complex patients.

The issues around airway management are valid and SOBA strongly advocates specific training in this area for all anaesthetists who care for patients with elevated BMI. Similarly, the problems highlighted surrounding the transfer of the patient with obesity are of significant concern to SOBA, and something which has been brought to the forefront of care during the COVID pandemic with many intubated patients requiring transfers. As a specialist society, we are currently formulating guidance for this high-risk area, which we hope will support our colleagues in their practice.

It is clear from the sections on complex scenarios that the authors share our concerns around specific training and research in management of the patient with obesity during emergency care. Despite obesity being a common comorbidity in the emergency care setting, there is a noticeable lack of research in this field.

It should also be remembered that this group of patients face a degree of fat-shaming, stigma and clinician bias which (in part) leads to healthcare avoidance and later presentations, resulting in more advanced disease and increased presentation to emergency departments [2].

In conclusion, SOBA UK welcome this review and agree with the need for improved guidance and organisational preparedness in this area. We would be happy to collaborate with other interested groups to improve safety for those living with obesity.
